# Knowledge, Attitude, and Practice towards Hepatitis B Infection Prevention and Screening among Indonesians

**DOI:** 10.3390/ijerph19084644

**Published:** 2022-04-12

**Authors:** Angga Dwiartama, Wahyu Fadzilla Nirbayati, Ernawati Arifin Giri-Rachman, Wardono Niloperbowo, Marselina Irasonia Tan, Anindyajati Anin

**Affiliations:** 1 School of Life Science & Technology, Institute Teknologi Bandung, Bandung 40132, Indonesia; wahyufadzilla@gmail.com (W.F.N.); erna@sith.itb.ac.id (E.A.G.-R.); wardono@sith.itb.ac.id (W.N.); marsel@sith.itb.ac.id (M.I.T.); 2 School of Pharmacy, Institute Teknologi Bandung, Bandung 40132, Indonesia; anin@itb.ac.id

**Keywords:** diagnostic kit, hepatitis B, PLS-SEM, screening, relationship

## Abstract

Hepatitis B has been one of the most prevalent infectious diseases in the world and specifically in Indonesia. Although the total conversion of hepatitis B virus (HBV) to chronic disease in Indonesia was reduced by 50%, the total number of hepatitis B cases increased by 2.5 times in 2021. Ineffective HBV immunization program in Indonesia prior to 1997 was addressed by the Ministry of Health through a more comprehensive HBV control, which, among others, involved Health Promotion to increase people’s knowledge and awareness towards hepatitis B infection prevention. In this regard, this study aims to identify the level of knowledge, attitude, and practice/behavior of the Indonesian population towards hepatitis B infection prevention and their willingness for screening, particularly in areas with high prevalence of hepatitis B. This study used a quantitative approach in looking at correlations between this set of knowledge, attitude, and practice, mainly by using Structural Equation Model (SEM) and SmartPLS 3.3.3 toolkit in SPSS. Through an analysis of online questionnaire results from over 400 respondents in four provinces (DKI Jakarta, West Java, DI Yogyakarta, and South Sulawesi), this study shows that respondents have a very high level of knowledge about hepatitis B; high level of positive attitude; and very high level of positive practice towards hepatitis B infection prevention. However, we also noticed some dissonances between the key aspects, namely that knowledge on hepatitis B correlates negatively with the behavior of the respondents and that the behavior also correlates negatively with their willingness for screening. In conclusion, we suggest that factors such as socio-economic context and prior informed knowledge on hepatitis B be considered to build a better strategy of Health Promotion and hepatitis B diagnostic screening among the population.

## 1. Introduction

The World Health Organization (WHO) estimates that 296 million people around the world were living with chronic hepatitis B infection in 2019, with 1.5 million new infections each year [1]. It is the major cause of acute and chronic liver disease and a global health problem [2]. In the Southeast Asian region, Indonesia is positioned as the second highest in terms of hepatitis B prevalence after Myanmar. Based on information from Indonesia’s Ministry of Health [3], the highest number of cases of hepatitis B was found in Java and Sulawesi Province.

The average prevalence of hepatitis B in Indonesia is 2.10%, showing an intermediate level of prevalence. In general, the western part of Indonesia has a low prevalence rate, while the eastern part has a medium prevalence rate, especially on the islands of Sulawesi, Maluku, and Papua, with an average prevalence of almost 5% [4]. Rate of transmission has been fast mainly due to blood transfusions containing the hepatitis B virus (HBV), with as many as 10 out of 100 people experiencing such a transmission. Although the total conversion of HBV to chronic disease has been reduced by 50%, the total number of hepatitis B cases in 2020–2021 (±2.7 million cases) compared to those in 2018–2019 (±1.1 million cases) have increased by around 2.5-fold. This condition caused Indonesia’s Ministry of Health to place a special focus on suppressing the spread of hepatitis B [5].

In addressing the global burden of hepatitis B virus (HBV) and its associated disease, the role of the HBV vaccine, which has been translated into an immunization program, has been pivotal [2]. Increased cases of hepatitis B have a close relationship with hepatitis B immunization (or lack thereof). In 1997, the government of Indonesia integrated a hepatitis B vaccination campaign into its National Immunization Program. After 1997, an early immunization program that was instituted by the government showed progress, whereby the younger population tends to be more resistant to HBV exposure as opposed to the generation born before 1997 (particularly in the age group between 20 and 40 years), which tends to have lower immunity [5]. Furthermore, a study by Muljono also illustrated that Indonesian people above 60 years old have lower than 55% resistance to hepatitis B [6].

This phenomenon occurs due to various factors, including the lack of public desire to carry out screening (due to people feeling healthy even without vaccination), which is exacerbated by limited availability of hepatitis B screening services [1]. Screening (i.e., a method to test healthy individuals for a disease before its symptoms appear) is not only able to detect hepatitis B virus but can also determine the success of the hepatitis B immunization program, as shown in the increased antibody protection against hepatitis B virus [7]. Regarding this, Indonesia’s Ministry of Health has since decreed Regulation No. 53 of 2015 that places particular emphasis on the comprehensive management of viral hepatitis B [8]. In Article 5 of this regulation, one of the key programs for the management of hepatitis B is Health Promotion, which involves increasing public knowledge on hepatitis B, its symptoms, transmission, and prevention measures. It is assumed that an increase knowledge will lead into an increase of public attitude and practice towards hepatitis B screening and prevention, thus reducing the prevalence of this disease in the population.

This study therefore aims at understanding the level of knowledge, positive attitude, and behavior of Indonesian people towards hepatitis B infection prevention. We aim at establishing a link between people’s knowledge of hepatitis B and their attitude and practice towards it in the hope that this study would inform the government on the effectiveness of its Health Promotion in the management of hepatitis B [8]. We focus on four provinces in Indonesia as samples for our study, each representing regions with high, low, and intermediate prevalence (DKI Jakarta, West Java, DI Yogyakarta, and South Sulawesi) [4]. These three variables (knowledge, attitude, and practice) have a close relationship with intention to screen as an effort towards prevention, which will be analyzed through SmartPLS [9].

## 2. Materials and Methods

### 2.1. Data Collection Method

This study uses a quantitative method followed by qualitative methods as enrichment. This type of research is observational with a cross-sectional approach through individual characteristics, level of knowledge, level of attitude, and level of community behavior regarding hepatitis B and towards the desire to carry out hepatitis B diagnostic screening. We conducted an online questionnaire survey to respondents from four cities: Bandung (West Java Province), Yogyakarta (DI Yogyakarta Province), Jakarta (DKI Jakarta Province), and Makassar (South Sulawesi Province). All respondents agreed to complete the questionnaires, and this study has been approved by the Human Ethics Committee. The population of this study consisted of people who had a history of hepatitis B along with the general population. The survey was conducted to a total of 400 respondents (100 respondents per region).

The operational definition of this research is shown in Table 1. For each of the variables (excluding the demographic characteristics), ten questions were provided to gain insights into the respondents’ knowledge of hepatitis B (its cause, symptoms, transmission, and prevention), positive attitude towards Hepatitis B infection prevention (vaccination, immunization program, avoiding blood transfusion, and screening), and behavior/practice towards hepatitis B prevention (have been vaccinated against HBV, have educated family on hepatitis B, etc.), resulting in a total of 46 questions. The list of questions used refers to the work of Balegha et al. [10], Lin et al. [11], and Hayati and Murtisiwi [12].

Because there is a correct answer for every question (which demonstrates the level of knowledge, positive attitude, and positive behavior), questions can be answered well if the average score is above 75% for each of the variables. This means that for knowledge (KNW), the indicator value is >1.5 (out of maximum value of 2 due to true and false answer, see Table 1); for attitude (ATT), it is >3.50 (out of 4); for practice (PRC), it is >1.5 (out of 2); and for intention to screen (ITP), it is >3.50 (out of 4). Further into the analysis, the closer a respondent gets to the maximum value, the higher their level of knowledge/attitude/practice/screening intention regarding hepatitis B prevention.

In order to obtain data that can support the main findings, we collected data using field observation techniques, surveys, interviews, and literature studies [13]. Data analysis was performed using a combination of descriptive statistics. The data obtained from the results of the questionnaire were processed using the help of the Statistical Package for the Social Science (SPSS) version and an add-on program of SmartPLS 3.3.3.

### 2.2. Data Analysis

**Figure 1:**
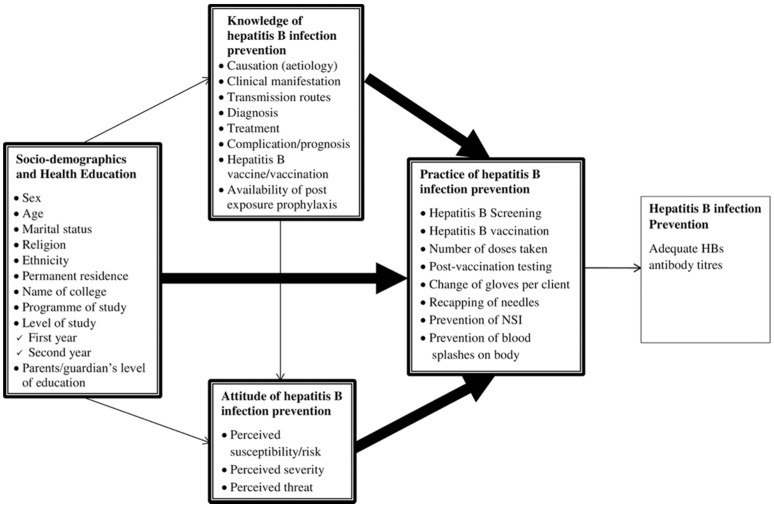
Reference model for looking at the relationship between Respondents’ Characteristics, Knowledge, Attitudes, and Practice of Hepatitis B infection prevention (Adapted with permission from: Balegha et al. [10]. PLoS ONE, Published by PLOS, 2021).

**Figure 2:**
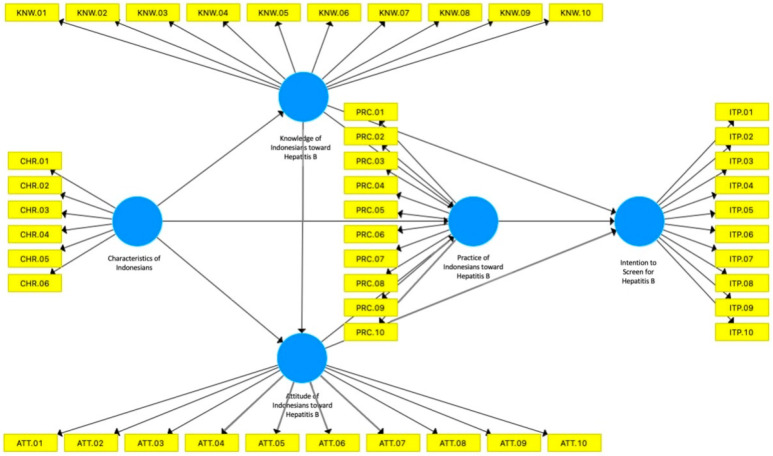
Initial conceptual model of Respondents’ Characteristics, Knowledge, Attitudes, Practice, and Intention for screening of Hepatitis B; note that yellow boxes correspond to the questions provided for each variable.

We developed our Structural Equation Model (SEM) based on a reference model proposed by Balegha et al. [10], as can be seen in Figure 1. In this model, the socio-demographic characteristics of the population, their knowledge of hepatitis B, and their positive attitude towards hepatitis B infection prevention will influence the increase of the practice of hepatitis B prevention among the population. In addition, there is also a correlation between each of the other variables: demographic characteristics and knowledge, demographic characteristics and attitude, as well as between knowledge and attitude. We developed this model further by integrating people’s willingness and intention to screen for hepatitis B, as inspired by Lin et al. [11]. The initial model of the relationship between respondents’ characteristics, knowledge, attitudes, practice, and respondents’ intention/willingness to do a screening diagnostic for hepatitis B, which integrates Balegha et al.’s and Lin et al.’s models, can be seen in Figure 2.

There are four stages carried out in the use of the SmartPLS 3.3.3 toolkit. The first stage is to develop a model according to the variables and inventory of the questionnaire. Secondly, we analyzed the factors through validity and reliability tests. This was followed by path analysis. Finally, the structural analysis of the model was carried out through the relationship strength test (R^2^), predictive relevance (Q^2^) test, and model fit test. The results of the model were then descriptively analyzed and discussed to provide insights into the common practice of Health Promotion in hepatitis B.

## 3. Results

### 3.1. Demographic Characteristics

There are six questions related to basic information conducted on respondents in four regions. These six basic information questions include gender (**CHR.01**), age (**CHR.02**), education level (**CHR.03**), occupation (**CHR.04**), income (**CHR.05**), and marital status (**CHR.06**), with the results shown in the following Table 2. The majority of respondents in this survey have the following characteristics: 59% are female; 44.75% are within the age range between 25 and 34 years old; 51.75% have a bachelor’s degree; 44% are working as an employee (in public or private sector); 33.25% have income below IDR 1,800,000 per month (equivalent to USD 120 per month, using an exchange rate of USD 1~IDR 15,000); and 57.25% of the respondents are married.

### 3.2. Knowledge Level on Hepatitis B among the Respondents

Based on Table 3, there are ten questions that reflect the level of public knowledge about hepatitis B. Results showed that respondents have a good level of general knowledge on hepatitis B except for questions 7 and 9, which indicate a poor level of knowledge among the respondents.

In general, respondents from the four geographic regions showed an average index of >85.00, which indicates a category of very high level of knowledge (index of 80.1–85 is considered high, 70.1–80 is medium, 60.1–70 is low, and <60 is very low in terms of level of knowledge). The review was continued by testing the difference in variance, which shows that there was no significant difference between the four regions. In general, people from the four regions showed an average index of 86.73, which in within the very high level of knowledge category.

### 3.3. Positive Attitude Level on Hepatitis B Prevention among Respondents

The results of the respondent’s attitude level towards hepatitis B in Table 4 shows a high level of positive attitude towards hepatitis B prevention among the respondents.

Based on the regional analysis, DI Yogyakarta has the highest average index of 85.30, with a very high level of attitude toward hepatitis B. Meanwhile, other areas showed an average index of <85.00, which can be categorized into a high level of attitude. Investigation of the variance test showed that there was no significant difference in the level of respondents’ attitudes towards hepatitis B. In general, the average index of all regions was 84.24, within a category of high level of attitude towards hepatitis B.

### 3.4. Practice/Behavior in Preventing Hepatitis B among Respondents

The tabulation of the question scores on the level of practice in Table 5 also shows a very high level of affinity towards positive behavior in hepatitis B prevention. Respondents from the four regions have an average practical level index of > 85.00, which means that the category is very high. The review was continued by testing the difference in variance, in which it was found that there was no real difference from the four regions to the level of practice. On average, people from the four regions showed an average index of 86.35, within the very high category. This shows that the public has a very high level of practice towards hepatitis B.

### 3.5. Willingness and Intention for Hepatitis B Screening

Investigation of the level of willingness of respondents to perform diagnostic screening for hepatitis B is presented in Table 6**.** Respondents’ results showed that questions 4, 6, 7, and 8 indicated a good level of desire for screening. Respondents from the Makassar area showed an average index of 82.55, which was categorized as a high level of desire for diagnostic screening, while the other three regions showed an average index of >85.00 (very high). Through the test of differences in variance, it was found that there were no significant differences between the four regions. Overall, it can be stated that people from the four regions showed a very high level of desire for hepatitis B diagnostic screening (85.25) as an effort for action of prevention.

### 3.6. PLS-SEM Model Relationship of Respondents’ Characteristics, Knowledge, Attitudes, and Practice of Hepatitis B toward Intention to Screen for Hepatitis B

The results of the questionnaire inventory were then modeled through the SmartPLS application. SmartPLS can basically perform factor analysis, path analysis, and model analysis simultaneously or concurrently [9]. If there is one relationship between variables that are not related, it can cause differences in the estimated values of the three analyses.

**Figure 3:**
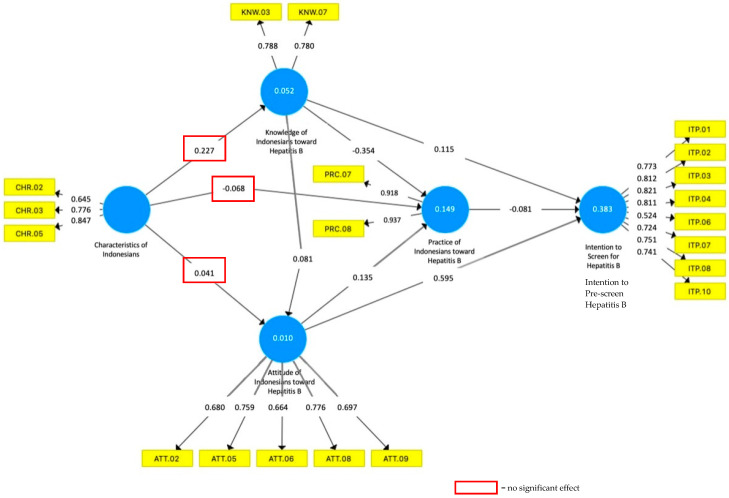
Final Model of the Relationship between Respondents’ Characteristics, Knowledge, Attitudes, and Practice of Hepatitis B toward Intention to Screen for Hepatitis B. Values are explained as follows: (1) values inside the lines connecting the blue circle (variable) and yellow boxes (indicators) shows the outer landing value of indicators; (2) values inside the blue circles show the R^2^ value, which indicates the endogenous latent variables; and (3) values connecting two blue circles show the path coefficient between two variables. Red boxes indicate no significant effect between the variables based on path analysis of the model.

The model in this study was compiled based on Balegha et al. [10] regarding their study of individual characteristics, knowledge of hepatitis B, as well as attitudes and on the prevention of hepatitis B with DDHB. The model was then refined by using another research concept that there is a relationship between knowledge, attitudes, and practice on other action intentions [11], as illustrated by the model in Figure 3, which has been proven valid and reliable.

The model in Figure 2 was developed through three development steps: (1) the development of initial conceptual model of PLS-SEM with SmartPLS (see Figure 2 above), (2) an identification of outer loading score of the initial model, and (3) the establishment of the final model according to the outer loading score. This model development refers to the work of Avkiran and Ringle [10]. Outer loading here is defined as score of indicators (i.e., questions to respondents) towards the given variable. If an outer loading of each specific indicator is lower than 0.50, the indicator would need to be excluded in order to build a strong correlational model.

As shown in Figure 3, three of the indicators of demographic characteristics (02. age, 03. education level, and 05. income level) are included in the final model. The demographic characteristics of participants are not scored in a way similar to the other variables but are treated based on the types of data representing each indicator, i.e., nominal, ordinal, interval, or ratio. Indicators with nominal data (gender, employment, and marital status) are coded with numbers but do not show any strong correlation to the other variables and thus are naturally eliminated. The ordinal/ratio data (age, education level, income level) are included in the model and show clear correlations with the other variables. This aligns with the logic that an increase in age, education level, or income level may have an effect on the level of knowledge, attitude, or practice towards hepatitis B infection prevention, as discussed further in Section 4.2.

Two indicators of knowledge, i.e., 03, that hepatitis B is not a hereditary disease, and 0.7, that hepatitis B is not transmitted through the digestive system, are included due to the questions being able to separate those with high level of knowledge about hepatitis B and the other respondents. There are five indicators for attitude, i.e., 02, attitude towards the efficacy of vaccine; 05, attitude towards healthy lifestyle; 06, attitude towards early immunization; 08, attitude towards screening; and 09, attitude towards willingness to pay for screening. Practice variable includes two indicators, namely 07, preventing other family members from being infected, and 08, willingness to self-isolate when exposed. Lastly, the intention/willingness for screening includes indicators such as 01–03; 06, choice of suitable diagnostic kits; 04, choice of friendly screening service; 07–08, trust in health workers; and 10, choice of proximity to screening facility.

### 3.7. Path Analysis of Model

One of the advantages of using SmartPLS in SEM analysis is that it can explain in detail the nature of the relationship or path with a level of significance [11]. Table 7 presents the value of the relationship from one variable to another directly. The range of values from the path is −1 to 1. If the path value is <0, it can be stated that the independent variable has a negative effect on the dependent variable. This applies the other way around if the path value is >0.

The significance test in SmartPLS can be obtained by bootstrapping Student’s *t*-test [9]. If the t-count is greater than the t-table, it can be declared significant. Through Table 7, there are six paths with a significance at the 95% confidence level, while the other three paths have no significance. In general, the nine pathways are categorized into two groups:

1. Significantly influence
  - Path 1 (+): Individual Characteristics to Hepatitis B Knowledge.
  - Path 5 (–): Hepatitis B Knowledge to Hepatitis B Practice.
  - Path 6 (+): Hepatitis B Knowledge to The Intention for Hepatitis B Screening.
  - Path 7 (+): Hepatitis B Attitude to Hepatitis B Practice.
  - Path 8 (+): Hepatitis B Attitude to The Intention for Hepatitis B Screening.
  - Path 9 (–): Hepatitis B Practice to The Intention for Hepatitis B Screening.
2. No significant effect
  - Path 2 (+): Individual Characteristics to Hepatitis B Attitude.
  - Path 3 (–): Individual Characteristics to Hepatitis B Practice.
  - Path 4 (+): Hepatitis B Knowledge to Hepatitis B Attitude.

### 3.8. Structural Analysis of Model

A review of the overall model value can be reviewed using three different tests. The strength of the relationship test, R^2^, is used to show the strength of the relationship of the variables used in the preparation of the model. The next test is predictive relevance, Q^2^, to measure the accuracy of the observation values generated by the model as well as parameter estimates. This was followed by the last test in the form of a fit model, which is intended to measure the quality of the model [14].

Based on the SmartPLS guidelines, the reference values for R^2^ are: weak (<0.25); medium (<0.50); strong (<0.75); and very strong (>0.75). The interpretation of the R^2^ value of the knowledge (0.052), attitude (0.010), and behavior (0.149) variables is categorized as having a weak relationship, whereas the desire for screening (0.383) has a moderate relationship category. The value of Q^2^ is positive for all variables arranged in the model. The reference value of Q^2^ in SmartPLS must be positive so that it can be said that the observation data on the model prepared are good [14].

The guideline value based used for the SRMR (Standardized Root Mean Squared) on the improvement model must be below 0.08 to be considered good, while the last parameter NFI (Normed Fit Index) is at 0.708 or 70.8%. The general model based on the SRMR (0.071) and NFI (0.708) can be stated as good or fit [14].

## 4. Discussion

### 4.1. Demographic Characteristics

The total results of the demographic characteristics of the respondents are similar when compared to the distribution of data in Indonesia. From Indonesia’s census data in 2020, the gender ratio in Indonesian population shows a rather equal ratio of 1:1, where 51% of population are men and 49% are women [15]. When viewed with the total data of respondents, the difference in the numbers obtained (41% men; 59% women) can be caused by various factors, such as the presence of respondents from an area that has a different gender ratio or other causes of selection bias [16,17].

Respondents from the four provinces (Table 2) stated that in general, they had completed their last education at the bachelor level. This is relatively different from the portrait of Indonesian Education 2020 [15], in which at least 90% of Indonesians have attended elementary school, while 60% have studied up to high school, and only about 20% of Indonesians are able to continue to higher education, especially at the bachelor’s degree level. Again, we identify this difference as part of our selection bias [17].

A total of 400 respondents from four areas affected by hepatitis B reported that the majority had main jobs as civil servants or employees at companies. However, the census data show that most Indonesians work in the agricultural and food sector [16]. The report further states that the proportion between employees working under the auspices of institutions and field workers in Indonesia is at a ratio of 1:1. This means that the distribution of jobs based on location in Indonesia is even. When compared with the survey results, employees at institutions (public or private) reached 44.0%, while the rest can be said to work elsewhere.

The income range question in the survey showed that in general, the monthly income is less than IDR 1,800,000 (equal to USD 120 based on the exchange rate of USD 1~IDR 15,000). Almost 50% of Indonesian people have income below the minimum wage [15]. This is caused by various factors, one of which is the lack of people’s ability to find decent and quality work to improve their standard of living. Another reason is that income in Indonesia has decreased due to the presence of the COVID-19 (coronavirus disease 2019) outbreak, which has had a major impact on the private sector, especially in the field of commerce and services [18].

Further review was carried out to compare the marital status of the respondents. A study by Kambuno et al. showed that about 54.5% of their respondents who were or had been infected with hepatitis B were married [19]. Meanwhile, the Bureau of Statistics Indonesia also shows that 59.98% of Indonesians are married [15]. When compared with the data in Table 2, we can see that the data on marital status reflect the general population in Indonesia as well.

### 4.2. Individual Characteristics to Hepatitis B Knowledge

Individual characteristics that are represented by indicators of age, last education, and income range showed a positive (0.227) and significant relationship with their level of knowledge. This means that each cumulative increase in the age range, last education, and income range by one unit will increase the cumulative knowledge level variable by 0.227, statistically speaking. Knowledge is obtained as people age due to their exposure of information but is also strengthened by the knowledge acquired in their formal education [20]. This means that it is true that the level of age and level of education might strengthen one’s knowledge about hepatitis B. However, as also shown by Hayati and Murtisiwi [12], there is a correlation between the level of education and the level of income (in a sense that level of education would provide a benefit to obtaining a better job, but a higher family income will guarantee a better education for the family members). This indicates that there is a complex relationship between education level, income level, and the ability of a person to access better knowledge on hepatitis B.

### 4.3. Hepatitis B Knowledge to Hepatitis B Practice

The level of knowledge towards hepatitis B has a path value of −0.354 when compared to the level of behavior/practice towards disease prevention or control. This value means that the level of public knowledge has a significant negative impact on the level of behavior. In other words, it can be concluded that the higher the level of knowledge, the lower their level of positive behavior towards hepatitis B infection prevention. This is counterintuitive to the linear relation between knowledge, attitudes, and behavior [20], whereby a better level of knowledge should provide a more positive attitude and behavior towards hepatitis B prevention. One possible reason why the knowledge of the respondents does not correspond to a better hepatitis B prevention practice is that knowledge itself does not guarantee a compliance towards certain practices, especially when people are inundated with other information as well. This situation can lead people to become less introspective to their health, as shown by Balegha et al. [10]. Other studies have also documented the dissonance between knowledge, attitude, and practice [21,22], showing the importance of socio-economic context in understanding why people behave in certain ways that are unrelated or even opposed to their knowledge and attitude.

### 4.4. Hepatitis B Knowledge to the Intention for Hepatitis B Screening

The result of path value from the knowledge level to the willingness for hepatitis B diagnostic screening is 0.115. The definition of the value is that the level of knowledge has a positive influence on the desire and willingness for diagnostic screening. The value is considered significant based on the Student’s *t*-test. The intention to act, as can be seen in our study, is influenced by the level of insight and knowledge [10,11]. Where the individual’s knowledge of hepatitis B is high, the intentions for engaging in a diagnostic screening that involves their trust in technology (high sensitivity and specificity kits) tend to be higher. Zibrik et al. [7] illustrated that an increase of public knowledge on hepatitis B (which was raised through a series of workshops for immigrants in Canada) is able to increase people’s willingness for screening. Furthermore, people with high level of knowledge tend to be selective about the health services that they use. In this regard, based on this study, we can infer that each cumulative increase in knowledge of one unit will increase interest in diagnostic screening by 0.115 units.

### 4.5. Hepatitis B Attitude to Hepatitis B Practice

Attitude level variables can be measured validly and reliably through five question indicators, including no. 2 (vaccines are considered effective), no. 5 (healthy lifestyles), no. 6 (early immunizations), no. 8 (maintaining family health), and no. 9 (screening with diagnostic kits). The path value obtained is 0.135. This value means that the level of attitude has a positive influence on the level of behavior. According to the basic concept of “Knowledge, Attitudes and Behavior”, a positive attitude will lead individuals to act in line with their level of behavior [20,22]. This also applies vice versa. This is in accordance with research conducted by Balegha et al. [10] on cases of hepatitis B prevention using the concepts of knowledge, attitudes, and behavior. Furthermore, normative attitudes towards hepatitis B should be able to provide follow-up reactions into more constructive preventive activities so that the spread of HBV can be more optimally suppressed.

### 4.6. Hepatitis B Attitude to the Intention for Hepatitis B Screening

Investigation on the respondents’ intention to screen for hepatitis B as correlated with their attitude level variable resulted in a value of 0.595, which implies a positive and significant correlation. Attitude, here, is seen as a response to a given stimulant [20]. People have high intentions for diagnostic screening based on their level of attitudes towards hepatitis B prevention. This is in accordance with the results of the study done by Lin et al. [11], who showed that the higher the public’s level of attitude, the higher their intention to take preventive measures. From this, it can be stated that the attitude level gives a positive increase of 0.595 units in the diagnostic screening intention if the cumulative level of attitude also increases by one unit.

### 4.7. Hepatitis B Practice to the Intention for Hepatitis B Screening

The analysis of the level of behavior/practice as correlated with the desire for diagnostic screening showed a negative (−0.081) and significant effect. It can be interpreted that every unit increase in the level of behavior will reduce the desire for screening by 0.081 units. This is, again, counterintuitive to the linear correlation between knowledge, attitude, and practice [20]. However, Lin et al. [11] explained that, in general, people act further as a result of previous actions. This means that if the preventive behavior is considered good, people would believe that they are not exposed to HBV. What is often missed is that HBV can be passed down through the biological mother of the individual [23]. Preventive measures are not always able to ensure that the person is protected from HBV. Detection through the diagnostic kit provides a more definitive picture because it is a predefined measuring instrument with greater accuracy.

### 4.8. Study Limitations

This study comes with a few limitations. Firstly, our choice of locations may not represent the whole populations in Indonesia, as each region has certain demographic and geographic characteristics that are different from others. Secondly, the survey was designed to recruit respondents regardless of their exposure to hepatitis B or closeness to family/friend who has the virus. This study, consequently, does not document knowledge, attitude, and practice of hepatitis B patients but rather of the general populations, with a small portion of the respondents potentially having a history of hepatitis B (see Table 5). Lastly, the online questionnaire survey may be inclined to a certain selection bias [17]. This selection bias may occur due to our specific use of online survey method, which pre-selected respondents on the basis of their affinity to mobile phones. This resulted in most respondents being of a certain age group, education level, and employment status. Although we acknowledge these three limitations, we nonetheless see the importance of the data variation on providing a certain depiction of the public knowledge, attitude, and practice on hepatitis B.

## 5. Conclusions

Demographic characteristics of most respondents in the affected areas (represented by DKI Jakarta, Bandung, DI Yogyakarta, and Makassar) are such that correspond to the general population in Indonesia (except for education level, which we considered a selection bias). This helps in furthering our understanding of the population’s response to hepatitis B, which shows that in general, people have high level of knowledge, attitude, and behavior towards hepatitis B prevention and willingness to screen for the virus. There are some inconsistencies in the correlation between knowledge and behavior, which makes sense when we consider the socio-economic context as well as the development of hepatitis B in these regions.

Over the 46 questions asked (all of which were considered indicators to the set variables of the initial model), a total of 20 indicators were later included in the final model. These indicators showed the type of factors that are important to the implementation of hepatitis B infection prevention and screening. Age, education level, and income level are three particular factors in the socio-economic profile that need to be taken into account if the government aimed to target specific groups of the population for its Health Promotion and Hepatitis B Screening campaign. Further criteria of diagnostic kits and health facilities, such as ergonomically design, effective, free, close to where the targeted population resides, provided by the government, and delivered by trusted health workers, are critical in the delivery of this screening campaign to increase the effectiveness of the future program. Stakeholders such as hospitals, midwives, the Indonesian Red Cross, and third-party health service facility laboratories will need to be involved in the process.

## Figures and Tables

**Table 1:** Operational Definition of Research.

Variables	Definition	Measurement	Results Interpretation	Data Scale
Demographic Characteristic (**CHR**)	Characteristics of the population	Six questions about gender, age, education level, employment status, income range, marital status	Categorized by each classified number	Nominal
Knowledge (**KNW**)	Public understanding of hepatitis B.	Ten questions about hepatitis B	1 = False 2 = True	Interval
Attitude (**ATT**)	Public response of hepatitis B	Ten questions about hepatitis B response	1 = Strongly Disagree 2 = Disagree 3 = Agree 4 = Strongly Agree	Interval
Practice (**PRC**)	Public action on hepatitis B	Ten questions about hepatitis B action	1 = Yes 2 = No	Ratio
Intention to Screening (**ITP**)	Public intention to prevent through screening	Ten questions about hepatitis B screening	1 = Strongly Disagree 2 = Disagree 3 = Agree 4 = Strongly Agree	Interval

**Table 2:** Demographic Characteristics of Respondents’ Profile.

Demographic Characteristics	DKI Jakarta	Bandung	DI Yogyakarta	Makassar	Total	
	n	n	n	n	n	%
Gender						
Male	47	44	29	44	164	41.00
Female	53	56	71	56	236	59.00
Age						
15–24	21	14	11	31	77	19.25
25–34	44	40	38	57	179	44.75
35–44	26	28	35	8	97	24.25
45–54	9	13	11	2	35	8.75
>55	0	5	5	2	12	3.00
Education Level						
Primary/High School	27	20	14	37	98	24.50
Diploma	4	10	11	5	30	7.50
Bachelor	50	52	57	48	207	51.75
Master	17	15	13	10	55	13.75
Ph.D.	2	3	5	0	10	2.50
Job						
Not Working	4	2	8	4	18	4.50
Housewife	8	16	15	12	51	12.75
Employee	45	47	41	43	176	44.00
Student	24	8	9	22	63	15.75
Others	19	27	27	19	92	23.00
Income Range						
<IDR 1,800,000	28	22	38	45	133	33.25
IDR 1,800,000–3,000,000	9	17	33	25	84	21.00
IDR 3,000,001–7,200,000	21	40	19	23	103	25.75
>IDR 7,200,000	42	21	10	7	80	20.00
Marital Status						
Married	56	61	56	56	229	57.25
Not Married	40	36	40	43	159	39.75
Others	4	3	4	1	12	3.00

**Table 3:** Knowledge level on hepatitis B among the respondents.

KNW	Question	DKI Jakarta	Bandung	DI Yogyakarta	Makassar	Overall
01	Hepatitis B is dangerous for humans	1.97	1.99	1.98	1.98	1.98
02	Hepatitis B is marked by yellowish skin	1.82	1.78	1.77	1.92	1.82
03	Hepatitis B is a hereditary disease	1.69	1.68	1.61	1.45	1.61
04	Hepatitis B is passed through pregnancy	1.66	1.62	1.71	1.63	1.66
05	Hepatitis B is marked by sore joints	1.72	1.73	1.68	1.81	1.74
06	Hepatitis B attacks the liver	1.96	1.96	1.94	1.88	1.94
07	HBV enters through digestion	1.38	1.35	1.3	1.18	1.30
08	Liver damage is a symptom of hepatitis B	1.91	1.9	1.85	1.89	1.89
09	Hepatitis B is transmitted through sex	1.48	1.54	1.47	1.44	1.48
10	Hepatitis B can be prevented	1.95	1.96	1.92	1.92	1.94
**Total Score**		**17.54**	**17.51**	**17.23**	**17.10**	**17.35**
**Average index**		**87.70**	**87.55**	**86.15**	**85.50**	**86.73**
**Level Category**		**Very High**	**Very High**	**Very High**	**Very High**	**Very High**
***p*-value (α = 5%)**		**0.966 (No Significant Difference)**				**-**

**Table 4:** Positive attitude level on hepatitis B prevention among respondents.

ATT	Question	DKI Jakarta	Bandung	DI Yogyakarta	Makassar	Overall
01	People need to get vaccinated	3.63	3.69	3.72	3.43	3.62
02	Vaccines are effective in preventing the transmission of hepatitis B	3.49	3.47	3.53	3.37	3.47
03	Avoid direct contact with sufferers	3.02	2.69	2.93	3.07	2.93
04	Willingness to examine oneself to health services	3.40	3.42	3.47	3.25	3.39
05	Healthy lifestyle prevents hepatitis B	3.60	3.64	3.61	3.42	3.57
06	Early immunization at birth	3.44	3.39	3.52	3.31	3.42
07	Refuse blood transfusions from patients	3.66	3.55	3.62	3.50	3.58
08	Maintain a healthy family environment	3.69	3.71	3.65	3.53	3.65
09	Screening check with diagnostic kit	3.33	3.33	3.34	3.21	3.30
10	Willing to pay the screening fee	2.70	2.78	2.73	2.94	2.79
**Total Score**		**33.96**	**33.67**	**34.12**	**33.03**	**33.70**
**Average index**		**84.90**	**84.18**	**85.30**	**82.58**	**84.24**
**Level Category**		**High**	**Very High**	**High**	**High**	**High**
***p*-value (α = 5%)**		**0.861 (No Significant Difference)**				**-**

**Table 5:** Practice/Behavior in preventing hepatitis B among respondents.

PRC	Question	DKI Jakarta	Bandung	DI Yogyakarta	Makassar	Overall
01	Have a history of hepatitis B	0.95	0.91	0.92	0.89	0.92
02	Have been vaccinated against hepatitis B	0.61	0.64	0.61	0.49	0.59
03	Hepatitis B in children is prevented through early immunization	0.97	0.95	0.97	0.88	0.94
04	Hepatitis B can be prevented through a healthy lifestyle	0.98	0.98	0.99	0.98	0.98
05	Willing to educate family	1.00	0.95	0.93	0.95	0.96
06	Willing to handle exposed family members	0.99	0.99	1.00	0.99	0.99
07	Prevent members’ contact with exposed persons	0.73	0.63	0.70	0.81	0.72
08	Willing to self-isolate if exposed	0.65	0.57	0.64	0.73	0.65
09	Request a new syringe in medical procedure	0.99	0.99	0.99	0.96	0.98
10	Dispose of B3 waste in an orderly manner	0.88	0.90	0.98	0.87	0.91
**Total Score**		**8.75**	**8.51**	**8.73**	**8.55**	**8.64**
**Average index**		**87.50**	**85.10**	**87.30**	**85.50**	**86.35**
**Level Category**		**Very High**	**Very High**	**Very High**	**Very High**	**Very High**
***p*-value (α = 5%)**		**0.980 (No Significant Difference)**				**-**

**Table 6:** Willingness and Intention for Hepatitis B Screening.

ITP	Question	DKI Jakarta	Bandung	DI Yogyakarta	Makassar	Overall
01	Choose an ergonomic diagnostic kit	3.52	3.62	3.46	3.32	3.48
02	Choose an effective and efficient diagnostic kit	3.52	3.63	3.48	3.32	3.49
03	Choose a diagnostic kit from the government	3.54	3.52	3.48	3.34	3.47
04	Love the friendly screening service	3.63	3.72	3.58	3.46	3.60
05	Willing to pay for a diagnostic kit	2.76	2.83	2.67	2.97	2.81
06	Option for free diagnostics	3.65	3.65	3.57	3.44	3.58
07	Trust the diagnostic kit recommended by health workers	3.56	3.53	3.54	3.39	3.51
08	Diagnostic kit demonstration by health personnel	3.58	3.67	3.59	3.30	3.54
09	Diagnostic kits are easy to get	3.23	3.11	3.14	3.19	3.17
10	Diagnostic kits available close to the community	3.50	3.54	3.56	3.29	3.47
**Total Score**		**34.49**	**34.82**	**34.07**	**33.02**	**34.10**
**Average index**		**86.23**	**87.05**	**85.18**	**82.55**	**85.25**
**Level Category**		**Very High**	**Very High**	**Very High**	**High**	**Very High**
***p*-value (α = 5%)**		**0.425 (No Significant Difference)**				**-**

**Table 7:** Path Analysis and Bootstrapping Student’s *t*-test.

No.	Path	Original Sample (O)	Sample Mean (M)	Standard Deviation (STDEV)	T Statistics (|O/STDEV|)	*p*-Values (α = 5%; 1.960)
1	CHR → KNW	0.227	0.235	0.047	4.819	0.000 *
2	CHR → ATT	0.041	0.037	0.057	0.706	0.480
3	CHR → PRC	−0.068	−0.070	0.046	1.474	0.141
4	KNW → ATT	0.081	0.084	0.051	1.573	0.117
5	KNW → PRC	−0.354	−0.353	0.049	7.275	0.000 *
6	KNW → ITP	0.115	0.115	0.040	2.885	0.004 *
7	ATT → PRC	0.135	0.135	0.048	2.803	0.005 *
8	ATT → ITP	0.595	0.599	0.034	17.605	0.000 *
9	PRC → ITP	−0.081	−0.081	0.041	2.003	0.046 *

* Indicates that significance at *p*-values < 0.05.

## Data Availability

The data presented in this study are available on request from the corresponding author. The data are not publicly available due to privacy issue (containing respondents’ information) and in accordance with the requirement of the Ethics Committee.
